# Action execution and observation in autistic adults: A systematic review of fMRI studies

**DOI:** 10.1002/aur.3291

**Published:** 2024-12-14

**Authors:** Sara Stillesjö, Hanna Hjärtström, Anna‐Maria Johansson, Thomas Rudolfsson, Daniel Säfström, Erik Domellöf

**Affiliations:** ^1^ Department of Psychology Umeå University Umeå Sweden; ^2^ Umeå Center for Functional Brain Imaging (UFBI) Umeå University Umeå Sweden; ^3^ Department of Health, Education and Technology Luleå University of Technology Luleå Sweden; ^4^ Department of Occupational Health, Psychology and Sports Sciences University of Gävle Gävle Sweden; ^5^ Department of Medical and Translational Biology Umeå University Umeå Sweden

**Keywords:** action execution, action imitation, action observation, autism, autism spectrum disorder, fMRI, motor

## Abstract

Motor impairments are common in individuals with autism spectrum disorder (ASD) although less is known about the neural mechanisms related to such difficulties. This review provides an outline of functional magnetic resonance imaging (fMRI) findings associated with execution and observation of naturalistic actions in autistic adults. Summarized outcomes revealed that adults with ASD recruit similar brain regions as neurotypical adults during action execution and during action observation, although with a difference in direction and/or magnitude. For action execution, this included higher and lower activity bilaterally in the precentral cortex, the parietal cortex, the inferior frontal gyrus (IFG), the middle temporal gyrus (MTG), the occipital cortex, and the cerebellum. For action observation, differences mainly concerned both higher and lower activity in bilateral IFG and right precentral gyrus, and lower activity in MTG. Activity overlaps between action execution and observation highlight atypical recruitment of IFG, MTG, precentral, and parieto‐occipital regions in ASD. The results show atypical recruitment of brain regions subserving motor planning and/or predictive control in ASD. Atypical brain activations during action observation, and the pattern of activity overlaps, indicate an association with difficulties in understanding others' actions and intentions.

## Lay Summary

Movement difficulties are common in autistic individuals, possibly at least partly due to problems with planning the required motor activities to achieve an action goal and monitoring this motor plan during action. Problems with performing actions in autism may also be related to problems with understanding the action of others. One way to increase the understanding of movement problems in autism, and the particularities that may be related to such problems, is to explore what goes on in the brain during action performance. In this article, we present an overview of studies from the last 20 years that have used functional brain imaging to investigate brain activity during execution, observation, and imitation of naturalistic actions (i.e., ordinary motor behavior in the physical world) in autistic adults compared with neurotypical adults. Summarizing the various study results, we found that autistic adults mainly showed brain activity in similar brain areas as neurotypical adults, although differing in magnitude and/or direction (higher or lower activity). This indicates atypical engagement of brain regions that are important for action‐related activities, such as movement planning and performance and accessing prior experience of movement in autistic individuals. Given the frequency of movement problems in autism, it is therefore of importance to continue investigating how these problems relate to brain activity. This will help to establish a reliable scientific basis for clinical recommendations and interventions, and potentially add to the understanding of autism in general.

## INTRODUCTION

Individuals with autism spectrum disorder (ASD) display atypical social communication and interaction, restrictive/repetitive thoughts and behaviors and insistence on sameness, contributing to functional limitations in everyday life (American Psychiatric Association, 2013). In addition to core symptoms of the diagnosis, impaired motor skills are also commonly observed (Coll et al., 2020). Impaired motor skills include reduced motor anticipation (Bäckström et al., 2021; Cattaneo et al., 2007; Zheng et al., 2019), atypical postural control (Wang et al., 2016), and reduced fine motor skills (Sacrey et al., 2014). It has even been argued that impaired motor skills should be entered as a core symptom of ASD (Bhat, 2021).

Despite a growing interest in motor deficits in ASD, few studies have used functional magnetic resonance imaging (fMRI) to target the underlying neural mechanisms related to atypical motor performance. As ASD is a pervasive neurodevelopmental condition (i.e., with symptoms across several different domains), the underlying neural mechanisms of impaired motor performance may also be an important foundation for other symptoms. Therefore, an increased understanding of the neurobiological underpinnings of atypical sensory‐motor behavior in ASD could also shed light on the broader ASD phenotype.

According to one popular theory, motor impairments in individuals with ASD may arise from abnormal recruitment of functional brain networks involved in motor planning and/or predictive control (Casartelli et al., 2018). In neurotypical (NT) populations, these anticipatory motor activities seem to engage frontoparietal and occipitotemporal regions (Gallivan et al., 2016; Säfström & Domellöf, 2018), but it is unclear if similar activations occur during motor planning and predictive control in ASD. Reduced predictive ability has further been proposed to influence core ASD symptoms involving social interaction (Cannon et al., 2021; Sinha et al., 2014). A possible link between the motor and social domains could be that prediction skills both influence the ability to physically interact with objects in the external environment and are critically involved in understanding the actions of others (Kilroy et al., 2022).

As suggested by the mirror neuron system (MNS) hypothesis, sensory information of other's action is coded as a motor representation by the MNS and is comparable to the internal representation formed when performing the same action (Rizzolatti & Fabbri‐Destro, 2008). The MNS includes the ventral premotor cortex, inferior parietal lobe (IPL), and inferior frontal gyrus (IFG) (Caspers et al., 2010; Molenberghs et al., 2012; Rizzolatti & Sinigaglia, 2016; Rizzolatti et al., 2014). A corresponding circuitry to the MNS is the proposed action observation network (AON), including activations in bilateral frontoparietal and posterior temporal regions during observation of other's action (Karakose‐Akbiyik et al., 2023). Notably, there is an overlap between the MNS and regions subserving motor performance, thus suggesting a possible overlap in neural activity between action execution and action observation. Similarly, in the AON framework, action execution and action observation are seen as two separate but overlapping networks (Condy et al., 2021). Corresponding recruitment between action execution and observation has been reported in the superior parietal lobule and dorsal premotor cortex, also involving additional activations in middle cingulate and somatosensory cortex (Marangon et al., 2011; Rizzolatti et al., 2014). In both the MNS and the AON framework, matching the representation of an observed action with stored experience of own action typically makes the behavior and intention of other's action comprehensible. Relatedly, both observation and imitation have been linked to a simulation circuit within the MNS that initiates an association between one's own action and action of other people (Rizzolatti & Sinigaglia, 2010). In addition, according to a large‐scale activation likelihood estimation meta‐analysis, there is a bilateral network for both action observation and imitation within premotor, parietal, and temporo‐occipital cortex (Caspers et al., 2010). Moreover, a broad meta‐analysis of neuroimaging studies investigating action execution, observation and imitation revealed activity in a similar network of premotor, parietal, and somatosensory regions across all actions, but with differences regarding recruitment of the putamen and cerebellum (Hardwick et al., 2018). Compromised predictive ability in ASD, however, may cause problems with understanding the “what” and “why” of actions performed by others (Cattaneo et al., 2007), and the intricacies of atypical motor mapping and motor‐based understanding in ASD warrant further investigation (Casartelli et al., 2018).

Most imaging studies involving autistic participants to date have focused either on functional connectivity based on resting‐state data or action observation and/or imitation tasks to evaluate abnormal brain recruitment in ASD. According to a recent comprehensive functional connectivity mega‐analysis (Ilioska et al., 2023), ASD is characterized by hypoconnectivity within sensory‐motor and attentional networks, and hyperconnectivity between the default mode network and the remaining brain, as well as between cortical and subcortical systems. Both the hypoconnectivity and the hyperconnectivity pattern were also linked to sensory processing difficulties and ASD severity. In keeping, a meta‐analysis of fMRI studies investigating action observation and imitation in ASD demonstrated hyperactivity in the anterior inferior parietal lobule (part of the MNS), possibly related to impaired sensory to motor remapping, and abnormal engagement of brain regions subserving visual processing, executive function, and social cognitive function (Yang & Hofmann, 2016). Thus, atypical brain processing related to motor execution, observation, and imitation share some commonalities within the MNS/AON. Still, it remains unclear if they should be regarded as parallel manifestations of fundamentally poor prediction and/or motor planning skills, or as distinctive traits of autism.

An additional finding in behavioral studies of movement‐related aspects of autism is large variability within and between individuals during motor performance (Noel et al., 2020). This variability could reflect impairments in feedforward and feedback control mechanisms, possibly subserved by the cerebellum (Mostofsky et al., 2009).

The main objective of the present review is to provide a comprehensive overview of fMRI findings related to motor performance during execution, observation and imitation of actions in adults with autism. While we recognize the importance of developmental perspectives and studies on children, this review focuses specifically on adults in order to examine brain activations patterns that persist beyond childhood and adolescence. Future reviews should address developmental trajectories, but this lies outside the scope of the current study. Two previous reviews have focused on fMRI studies investigating ASD compared with NT individuals across various domains, including motor action (Philip et al., 2012), and in relation to action observation (Chan & Han, 2020). Here, we specifically focus on differences in brain activations during execution, observation, and imitation of naturalistic actions between adults with and without ASD. Naturalistic actions refer to ordinary motor behavior in the physical world, such as the direct use of gestures or interaction with objects (i.e., not animations, point‐light displays, cartoons, still images, or similar). We also provide a description of the overlap in brain activations across regions where autistic adults differ from NT ones, as identified by the included studies. Based on previous literature, we expect to find the articles included in this review to report findings associated with engagement of frontoparietal and occipitotemporal cortical regions, the MNS/AON and the cerebellum. Regarding participants with ASD, we hypothesize that activations will be similar in location but deviating in magnitude and/or direction.

## METHODS

We followed the preferred reporting items for systematic review and meta‐analysis (PRISMA) guidelines for reporting this systematic review (Page et al., 2021).

### Search strategy

The database search included international scientific studies written in English published in peer‐reviewed journals between years 2000–2023 from the PubMed database and Web of Science. The keyword combination used for database search was “(Autism OR Asperger) AND (fMRI OR functional magnetic resonance imaging) AND (Action OR Movement OR Motor OR premotor).” The database search was performed on April 17th 2023.

### Inclusion/exclusion criteria

For inclusion, the articles found with this search strategy should focus on adults with ASD versus NT adults. A formal psychiatric ASD diagnosis was required (such as preestablished from DSM‐V or comparable) and a clear framework for execution/observation/imitation of a naturalistic motor task had to be reported together with imaging results. Only studies reporting task‐related fMRI were included.

Criteria for exclusion were if participants with ASD had been grouped with participants with other developmental conditions (e.g., intellectual disability, cerebral palsy, and developmental coordination disorder). Articles that focused on motor tasks that only involve speech production or other activation related to language were also excluded. Additionally, all single‐case studies were excluded.

### Study selection

After removal of duplicates from the search, two authors (HH, SS) removed records that was clearly out of scope by screening titles. All authors then independently screened titles/abstracts of the identified records against the inclusion/exclusion criteria. Next, a second round of screening was performed, where members of the review team screened each other's selection once more to improve reliability. All authors met for a final decision process to discuss potential uncertainty related to inclusion/exclusion before the next step of the selection process. The same process was then used for the full text screening. Screening of additional records such as reference lists for the included articles was performed by three independent authors (ED, HH, SS) using the same procedure as for the database search. The review process of the final full‐text articles related to data extraction and analysis was performed by the same three authors of the review team.

### Data extraction and analysis

The synthesis of results was conducted by considering the research question (Focus: Execution/Observation/Imitation). The specific method that was used (behavioral and fMRI paradigm) was compiled, together with results and conclusions. The fMRI design was reported individually for each study. For fMRI results, functional localization in terms of BOLD activity associated with ASD versus NT for Execution/Imitation/Observation. Overlaps in brain activity between domains were summarized based on visual inspection of reported brain regions (*x*, *y*, *z* coordinates in Montreal Neurological Institute Space and Broadmann area). Note that there is a variety of statistical procedures and thresholds between the included studies, forming the basis of the reported brain regions of interest. Thus, the magnitude of reported results and size of the clusters differ between studies. Still, in each study, the same procedures were used for both the ASD and NT groups and, as such, have been used in the comparison. Data related to descriptive characteristics of the included studies focused on (1) Authors and year of publication; (2) Citations/Impact of each publication; (3) Demographic information of ASD and NT participants (age, sex, diagnosis, intelligence quotient [IQ], and handedness).

## RESULTS

**FIGURE 1:**
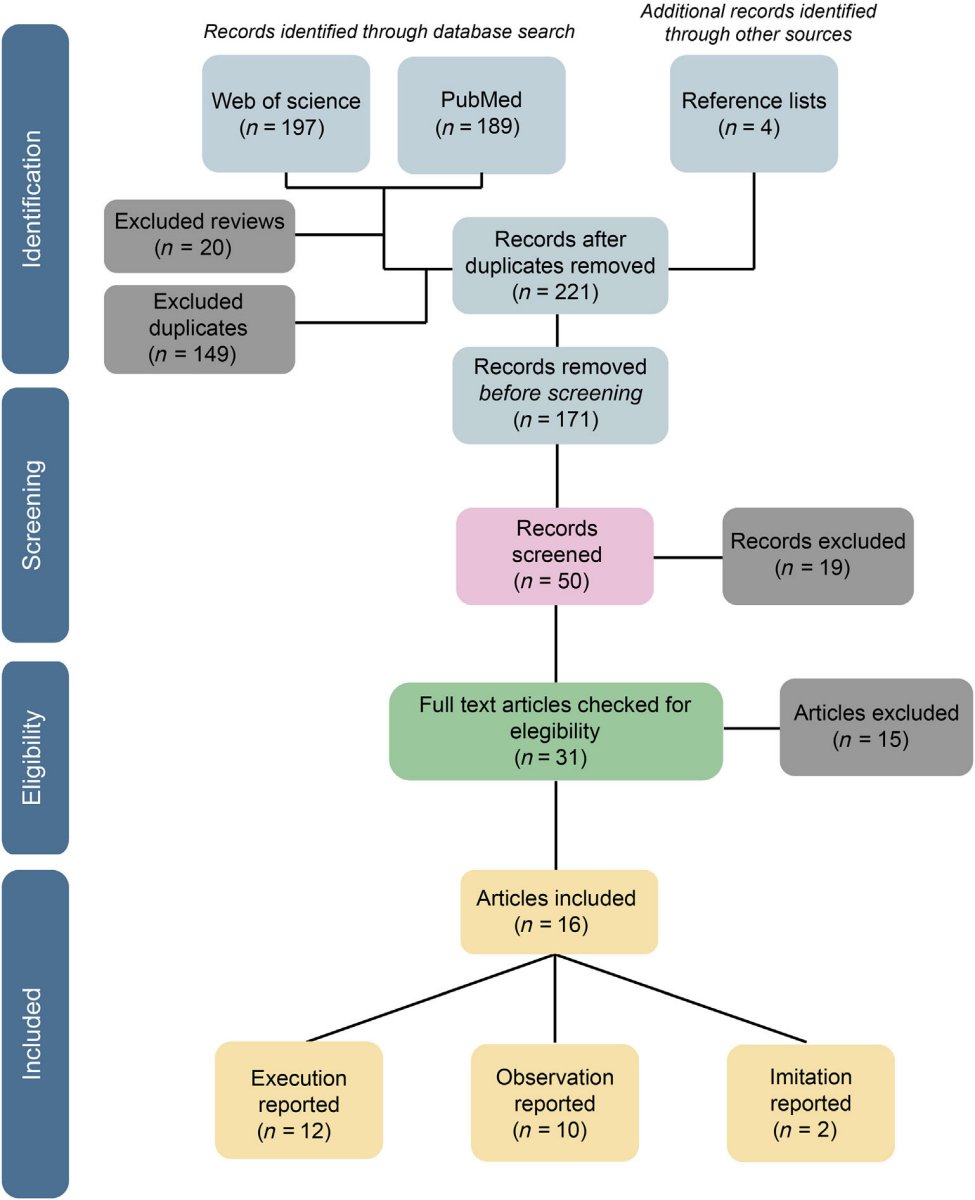
Flowchart diagram for the systematic review following the PRISMA guidelines.

After removing duplicates and reviews, our search identified 221 studies. Studies clearly out of scope for the present review were removed (*n* = 171), resulting in 50 studies eligible for screening of titles/abstracts. Having done this, 31 studies were identified as appropriate for full‐text screening. Of these 31 studies, 16 met the inclusion/exclusion criteria and were included in the review. The selection process is illustrated in Figure 1.

Table 1 gives an overview of descriptive characteristics for each article extracted from its respective method and result sections. The main results from each individual article are reported in Table 2.

**TABLE 1:** Group participant characteristics in the 16 reviewed studies.

Study		*N*	*M*_age_ (range)	Sex (M/F)	Diagnosis	*M*_IQ_ (SD)	Handedness
Studies focusing on action execution							
Allen and Courchesne (2003)^1^	ASD	8	26.89 (14–38)	7/1	DSM‐IV	85 (11.95)	62.5% RH
	NT	8	26.77 (13–39)	7/1	‐	113.75 (9.21)	62.5% RH
Allen et al. (2004)^1^	ASD	8	26.89 (14–38)	7/1	DSM‐IV	85 (11.95)	62.5% RH
	NT	8	26.77 (13–39)	7/1	‐	113.75 (9.21)	62.5% RH
Lepping et al. (2022)	ASD	27	18.4 (9–35)	24/3	DSM‐IV, ADI‐R, ADOS	Verbal: 102 (19.7) Nonverbal: 100.8 (19.6)	92.6% RH
	NT	30	18.9 (10–35)	18/12	‐	Verbal: 112.7 (15.6) Nonverbal: 107.8 (13.1)	86.7% RH
Müller et al. (2001)^2^	ASD	8	28.4 (15–41)	8/0	DSM‐IV, CARS, ADI‐R	86.5 (11.4)	62.5% RH
	NT	8	28.5 (21–43)	8/0	‐	>70 (N/A)	62.5% RH
Müller et al. (2003)^2^	ASD	8	28.4 (15–41)	8/0	DSM‐IV, CARS, ADI‐R	86.5 (11.4)	62.5% RH
	NT	8	28.5 (21–43)	8/0	‐	>70 (N/A)	62.5% RH
Müller et al. (2004)^2^	ASD	8	28.4 (15–41)	8/0	DSM‐IV, CARS, ADI‐R	86.5 (11.4)	62.5% RH
	NT	8	28.5 (21–43)	8/0	‐	>70 (N/A)	62.5% RH
Unruh et al. (2019)	ASD	20	21.11 (N/A)	18/2	DSM‐V, ADI‐R, ADOS	Verbal: 110.76 (18.61) Performance: 110.59 (15.13)	HLI 0.76
	NT	18	22.62 (N/A)	16/2	‐	Verbal: 118.69 (12.47) Performance: 117.77 (11.04)	HLI 0.93
Studies focusing on action observation							
Cole et al. (2019)	ASD	20	29.8 (N/A)	12/8	DSM‐V	N/A	95% RH
	NT	20	29.6 (N/A)	12/8	‐	N/A	95% RH
Grèzes et al. (2009)	ASD	12	26.6 (18–56)	10/2	DSM‐IV	102 (20.6)	N/A
	NT	12	21 (N/A)	12/0	‐	119 (6.6)	N/A
Marsh and Hamilton (2011)	ASD	18	33 (N/A)	12/6	ADOS	Verbal: 112.9 (16.6) Performance: 104.4 (18)	N/A
	NT	19	32.2 (N/A)	11/8	‐	Verbal: 112.5 (13.8) Performance: 113.4 (13.9)	N/A
Perkins et al. (2015)	ASD	12	18.5 (N/A)	12/0	DSM‐IV	N/A	N/A
	NT	12	19.75 (N/A)	12/0	‐	N/A	N/A
Schütz et al. (2020)	ASD	22	18.6 (N/A)	22/0	ADOS, ADI‐R, ICD‐10	104.73 (17.41)	86.4% RH
	NT	25	20.41 (N/A)	25/0	‐	106.48 (18.72)	88% RH
Studies focusing on action execution and observation							
Dinstein et al. (2010)	ASD	13	27.4 (19–40)	13/0	ADOS, ADI‐R	110 (range: 95–128)	84.6% RH
	NT	10	27.4 (21–35)	5/5	‐	N/A	80% RH
Martineau et al. (2010)	ASD	7	23 (19–31)	7/0	N/A	93.29 (range: 85–113)	100% RH
	NT	8	23.25 (19–31)	8/0	‐	N/A	100% RH
Studies focusing on action imitation							
Okamoto et al. (2014)	ASD	19	24.8 (N/A)	18/1	DSM‐V, DISCO	104.3 (15.5)	94.7% RH
	NT	22	24.2 (N/A)	20/2	‐	114.5 (8.1)	95.5% RH
Poulin‐Lord et al. (2014)	ASD	23	19.8 (14–30)	20/3	DSM‐IV, ADI‐R, ADOS	100.3 (10.48)	HLI 62.35 (range: −80–100)
	NT	22	22.6 (15–35)	19/3	‐	107.3 (12.51)	HLI 74.05 (range: −87.5–100)

*Note*: *N*, number of participants; *M*_age_, mean age; *M*_IQ_, mean intelligence quotient; ASD, autism spectrum disorder; NT, neurotypical; M, male; F, female; N/A, information not available; DSM‐IV/V, diagnostic and statistical manual 4th/5th edition; ADI‐R, autistic diagnostic interview revised; ADOS, autism diagnostic observation schedule; CARS, childhood autism rating scale; ICD‐10, international statistical classification of diseases and related health problems 10th revision; DISCO, diagnostic interview for social and communication disorder; RH, right‐handed; HLI, hand laterality index (ranging from −1/−100, completely left‐handed, to 1/100, completely right‐handed). ^1,2^Studies that appear to include the same participant sample.

**TABLE 2:** Experiment overview, behavioral findings, and key brain regions involved related to ASD in the 16 reviewed studies.

Study	Citations^a^	Overview of experiment		Behavioral findings	Key brain regions involved
Studies focusing on action execution					
Allen and Courchesne (2003)	258	Aim To investigate how cerebellar pathology impacts cognitive‐ and motor function within the cerebellum in ASD	Methods GE Signa 1.5T system Block design Participants performed a simple motor task over four blocks, pressing a button on a joystick with the thumb of dominant hand at a self‐paced speed	No significant group difference related to mean number of button presses (ASD = 75.3, SD = 18.8; NT = 58.9, SD = 23.5)	Motor task > Rest Significantly greater extent and magnitude of activation in right superior hemisphere lobule VIIA in ASD
Allen et al. (2004)	145	Aim To examine the dynamics of simple motor activation in ASD focusing on the relationship between structural volume and functional activity during motor performance	Methods GE Signa 1.5T system Block design Participants performed a simple motor task over four blocks, pressing a button on a joystick with the thumb of dominant hand at a self‐paced speed	No significant group difference in frequency of button presses (ASD = 66.81, SD = 8.5; NT = 57.35, SD = 25.0)	ROI × Diagnosis Significant interaction between magnitude in the anterior hemisphere ipsilateral to the moving hand and ASD Association structure and function Strong inverse association in right cerebellum (*r* = −0.74) and right superior lobule VIIa (*r* = −0.88) between anatomy and function, with increased activation in ASD
Lepping et al. (2022)	8	Aim To investigate visuomotor network activation and functional connectivity during precision gripping in ASD	Methods 3T GE scanner Block design Participants performed alternating force blocks (pressing a transducer to adjust a force bar and maintain force) and rest blocks (passive viewing of static force bar)	No significant group difference in either mean force or age‐associated effects ASD displayed increased force variability (especially at high gain) and reduced force entropy	Brain activation Increased activation in ASD at high gain in bilateral supplementary motor area, bilateral superior parietal lobule, contralateral (left) middle frontal gyrus Functional connectivity Reduced connectivity between ipsilateral (right) inferior parietal lobule and contralateral (left) ventral premotor cortex in ASD during action and rest Reduced connectivity between right inferior parietal lobule and left putamen during force compared with rest in ASD Increased connectivity with age in ASD compared with NT in relation to right/left cerebellar Crus I and right cerebellar lobules V/VI
Müller et al. (2001)	136	Aim To investigate abnormalities in cerebral organization underlying movement problems in ASD	Methods GE Signa 1.5T system Block design Participants performed alternating task blocks (pressing a button with index finger following a blue dot rhythmically flashing on the index finger of a still image of a hand) and control blocks (passive viewing of the blue dot without any movement)	No significant group difference in either mean number of button presses or temporal precision (SD of intervals between presses) Increased variability in ASD noted	Brain activation Increased activation in ASD in bilateral parieto‐occipital and contralateral prefrontal areas, and contralateral posterior middle temporal region Additional activation in contralateral superior temporal lobe, and deactivation of contralateral inferior frontal and insular cortices, for ASD only Individual variability Greater spatial variability of activations across individuals with ASD
Müller et al. (2003)	130	Aim To investigate abnormalities in frontal and parietal activation and variability during digit‐sequence learning in ASD	Methods GE Signa 1.5T system Block design Participants performed six‐digit sequences (pseudo randomized or regular) of finger movements prompted by visual guidance (blue dot rhythmically flashing on the fingers of a hand diagram) and control conditions (simple or complex visuomotor motor execution)	No significant group difference in mean number of response time during experimental conditions Significantly higher error rate in ASD than NT Increased variability in ASD noted No significant group difference in mean number of finger presses and error and response time decreases over trials within task blocks during control conditions	Pseudorandomized condition Increased activation in ASD in bilateral parietal lobes (inferior/posterior), bilateral premotor area, right medial frontal area, and superior frontal gyri Additional activation in left inferior and middle frontal region for ASD only Regular condition ASD displayed elevated activation in mainly left‐sided frontal (anterior to premotor cortex) and parieto‐occipital (inferior/posterior) regions Additional activation in bilateral parietal, premotor, and interior frontal areas, left prefrontal area, occipital cortex and right middle temporal gyrus for ASD only Individual variability Across both experiments, significant group difference (ASD > NT) in spatial variability (left and right premotor and superior parietal cortex)
Müller et al. (2004)	71	Aim To investigate disturbances in the typical pattern of learning‐stage‐specific frontal activity in ASD during visuomotor learning	Methods GE Signa 1.5T system Block design Participants performed eight‐digit sequences (regular) of finger movements prompted by visual guidance (blue dot rhythmically flashing on the fingers of a hand diagram), and a control condition (simple motor execution with index finger according to blue dot pace)	No significant group difference in response time Significantly higher number of errors in ASD than NT No significant group difference in learning‐related changes from early to late stages in terms of error rate and response time (significant decrease in response time for both groups over stages)	Brain activation Increased activation in ASD in bilateral inferior/anterior area 6, and primary motor cortex during early learning Increased right‐sided activation in precuneus for ASD (left‐sided for NT) Increased activation in ASD in right pericentral and premotor cortex during late learning Learning stage differences for ASD only in medial frontal areas, and mainly right visual and posterior cortices (early > late), and in right pericentral and premotor cortex, bilateral middle frontal gyrus and bilateral visual cortex (late > early)
Unruh et al. (2019)	15	Aim To characterize the links between visually guided precision gripping and brain activation in ASD	Methods 3T Phillips Achieva scanner Block design Participants performed alternating force blocks (pressing a transducer to adjust a force bar and maintain force) and no‐force blocks (passive viewing)	No significant group difference in mean force Elevated force SD for ASD, but not significant	Brain activation Increased activation in left putamen and left cerebellar VIIb in ASD Association diagnosis & activation Higher clinical ratings of ASD severity (ADOS) associated with greater activation of right precuneus, and higher ratings of repetitive behaviors (RBS‐R) with greater activation of left cerebellar lobule VIIb
Studies focusing on action observation					
Cole et al. (2019)	21	Aim To determine whether behavioral difficulties in mentalizing in ASD can be explained by changes in functional connectivity between the mentalizing and mirror system	Methods 3T GE HD excite MRI scanner Block design Participants performed a mentalizing task (indicate type of action from videos of clumsy or spiteful actions) and a nonmentalizing task (indicate type of action from videos of successful or unsuccessful clumsy actions)	No significant group difference in number of correct responses in either task The level of autistic traits in both ASD and NT groups predicted the number of correct responses on the mentalizing task, but not the nonmentalizing task	Connectivity analysis Significant interaction between mentalizing system region (dorsomedial prefrontal cortex, temporoparietal junction, orbitofrontal cortex), task, and group: higher levels of functional connectivity between dorsomedial prefrontal cortex and the mirror system region (inferior frontal gyrus, inferior parietal lobe) in the NT group compared with ASD Association traits and connectivity Higher level of autistic traits across all participants predicted reduced functional connectivity in dorsomedial prefrontal cortex and inferior frontal gyrus during the mentalizing task
Grèzes et al. (2009)	73	Aim To investigate activations in brain regions related to action perception and recognition of emotional meaning in ASD	Methods 3T whole‐body imager Event‐related design Participants viewed videos of people performing the simple action of opening a door with various outcomes	N/A	Conjunction analysis In ASD and NT, respectively, for dynamic and static stimuli: a common distributed network including superior temporal sulcus, intraparietal sulcus, precentral gyrus, and dorsal inferior frontal gyrus No significant group difference in automatic activation of motor representations during action perception. Reduced activation of amygdala, inferior frontal gyrus and premotor cortex in ASD in relation to fearful gestures
Marsh and Hamilton (2011)	83	Aim To investigate brain responses in social brain regions in ASD when observing rational (mirror components) and irrational (mentalizing components) hand actions	Methods 3T Phillips Achieva scanner Block design and event‐related repetition suppression Participants viewed videos corresponding to five conditions: rational action, rational action with barrier, irrational action, irrational action with barrier, and moving shapes	Significant group differences (ASD < NT) in mental state attribution and action comprehension (particularly intransitive social action)	Brain activation No significant group difference in response profiles in the parietal component of the mirror system (left anterior intraparietal sulcus) when viewing hand actions compared with moving shapes Evident group differences in brain activation in regions outside the mirror system in response to observing action (posterior middle cingulate cortex, supplementary motor area, fusiform cortex) Repetition suppression No significant group differences in activation related to selectivity to action goals. Both groups encoded goal of observed hand action in same brain regions (left anterior intraparietal sulcus mainly) with same trial‐to‐trial repetition suppression Observing irrational actions Compared with viewing rational actions: no significant group difference in activation of right anterior intraparietal sulcus. No differential activation of medial prefrontal cortex in ASD
Perkins et al. (2015)	29	Aim To investigate if ASD display increased BOLD response in frontal, parietal and temporal mirror neuron areas during observation of hand‐based gestures	Methods 3T Siemens Tim Trio scanner Block design Participants viewed experimental videos of hand‐object, hand‐mouth, hand‐communicative, and hand directive actions, alternated with control videos (motionless hand, expressionless face)	N/A	Brain activation No significant group differences when comparing activity in mirror neuron regions during action observation and baseline Increased BOLD signal in right premotor cortex (BA6), bordering precentral gyrus in ASD Increased activation in ASD in right frontal regions (rostral anterior cingulate cortex and medial frontal gyrus) during action observation
Schütz et al. (2020)	2	Aim To investigate neural activations during observation of communicative and noncommunicative actions in ASD	Methods 3T Siemens Allegra Block design Participants observed videos presenting actions of different intention (communicative/noncommunicative) and orientation (first‐person/third‐person)	No significant group difference in hit rates Distractor task: ASD significantly slower reaction times than NT for identifying female actor (button press) across all conditions	Brain activation Across all conditions and stimuli, NT showed higher activation in bilateral middle temporal gyrus than ASD Decreased activation in right premotor cortex in ASD (increased activation in NT) during observation of first‐person communicative actions. No changed activation in either group for observation of third‐person interactions
Studies focusing on action execution and observation					
Dinstein et al. (2010)	110	Aim To investigate movement selective responses in the MNS during observation and/or execution of hand movements in ASD	Methods 3T Allegra MRI scanner Block design Participants observed and executed blocks of still images of hand postures in repeat (single hand posture) or nonrepeat (six different hand postures)	N/A	Movement observation No significant group difference in fMRI response amplitudes in all ROIs Movement execution No significant group difference in fMRI response amplitudes in all ROIs Individual variability Significantly increased within‐subject variability (SD) in ASD for repeat and/or nonrepeat in several ROIs during both observation and execution No significant difference in SD between repeat and nonrepeat within the ASD group
Martineau et al. (2010)	84	Aim To investigate neural activity during observation and execution of human movements in ASD	Methods 1.5T Signa GE scanner Block design Participants either viewed a video of a hand performing a flexion‐extension movement at a rate of 1 Hz, or performed flexion‐extension at the same rate with their own hand while viewing still image of hand	N/A	Brain activation No significant group difference for either execution > rest, observation > execution or execution > observation For observation > rest, elevated activation of bilateral inferior frontal gyrus (pars opercularis) in ASD Additional activity in right cerebellum apart from left motor area (central sulcus) during execution > rest for ASD only Activation of right motor area (central sulcus), bilateral inferior parietal lobule (supramarginal gyrus) and bilateral occipital regions in ASD only for observation > rest ASD only showed greater activation of left central sulcus and right cerebellum for execution > observation
Studies focusing on action imitation					
Okamoto et al. (2014)	13	Aim Compared with NT, what is the effect of ASD on neural networks (activity in the extrastriate body area, EBA) underlying social contingency detection	Methods 3T GE Sigma Horizon imager Block design Participants made finger gestures indicating numbers 1 to 5 according to instructions, while observing videos of recorded finger gestures. Congruency and order of action/action observation were manipulated	No significant group difference in behavioral performances	Brain activation Reduced contingency effect in the left extrastriate body area in ASD when self‐action was imitated by recorded action No significant group difference in congruency effects within the extrastriate body area for imitating the recorded action
Poulin‐Lord et al. (2014)	27	Aim To investigate whether there is increased interindividual variability in localization, intensity and size of brain activations within primary and associative areas of visual and motor modalities in ASD	Methods 3T Siemens Tim Trio scanner Block design A visuomotor imitation task with 15 hand gestures was used, where participants observed and then imitated gestures with both left and right hand	N/A	Individual variability Increased interindividual variability in localization of activation in ASD in left associative visual areas (BA18‐19) and superior parietal cortex (BA7), independent of anatomical gray matter variability. No significant group difference regarding variability in intensity or size of brain activations Whole brain task‐related activity Left hand: increased activation in ASD in left lingual gyrus (BA19) Right hand: no significant group difference in regional activity Left + Right hand: increased activation in ASD in a range of regions subserving visual and motor processing (bilateral middle occipital gyrus, left cuneus, bilateral lingual gyrus, bilateral precuneus, right medial frontal gyrus, right superior frontal gyrus, and bilateral superior parietal lobule) ASD only: increased use of middle occipital gyrus and inferior semi‐lunar lobule, nodule and caudate (NT only: frontal regions)

^a^ Number of citations extracted from Web of Science December 14, 2023.

Abbreviations: ADOS, autism diagnostic observation schedule; ASD, autism spectrum disorder; BA, Brodmann area; BOLD, blood oxygen level dependent; N/A, information not available; NT, neurotypical; RBS‐R, repetitive behavior scale revised; ROI, region of interest; T, tesla.

### Included articles

**FIGURE 2:**
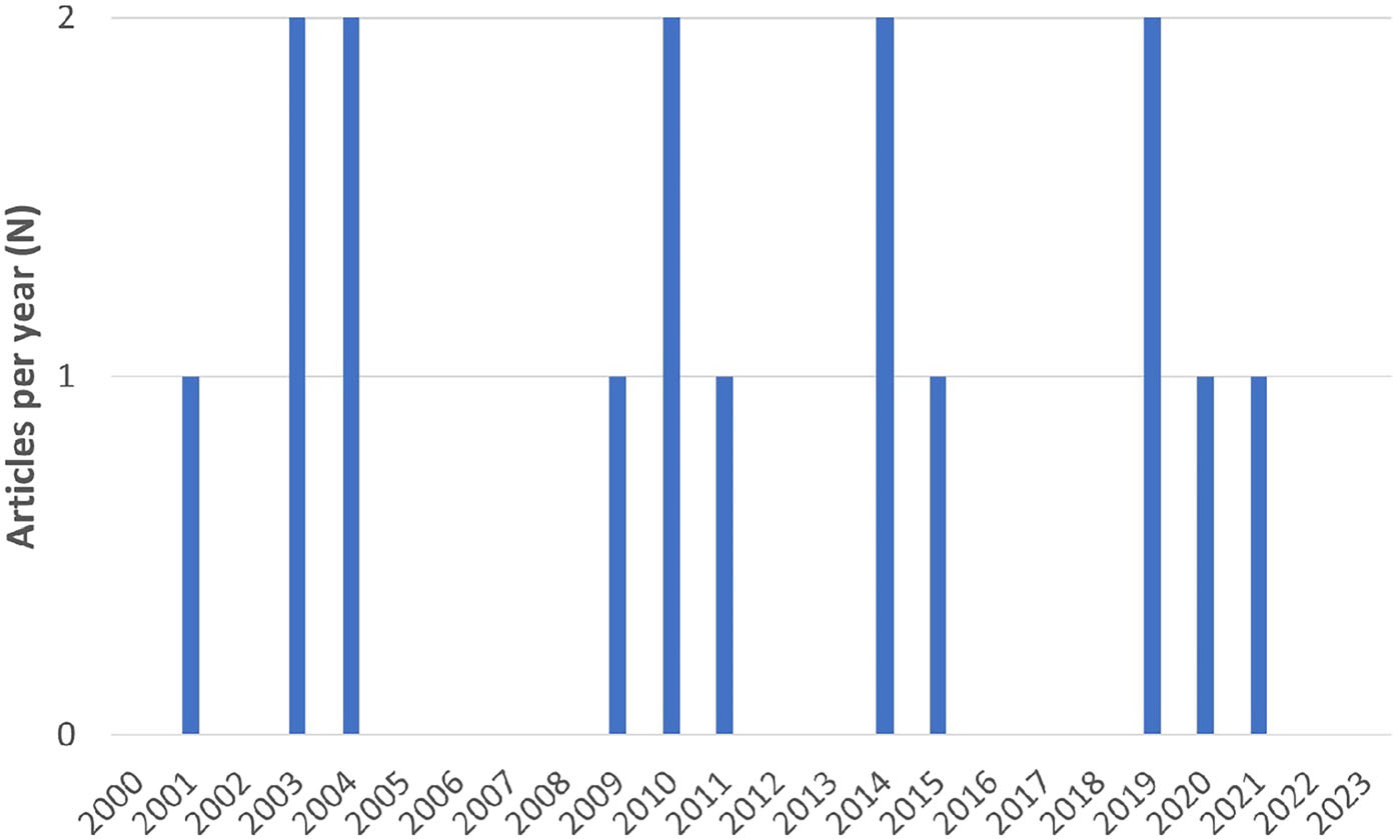
The distribution of publications/year for the 16 included articles between year 2000 and 2023. The number of publications is displayed on the *y*‐axis and publication year is displayed on the *x*‐axis.

The included articles (*n* = 16) were published between years 2001 and 2021 across 12 journals, with a scattered distribution of publications across years (see Figure 2). Eight studies (50%) originate from the US, two from the UK, two from France, one from Germany, one from Australia, one from Japan, and one from Canada.

Ten articles (Cole et al., 2019; Dinstein et al., 2010; Grèzes et al., 2009; Lepping et al., 2022; Marsh & Hamilton, 2011; Okamoto et al., 2014; Perkins et al., 2015; Poulin‐Lord et al., 2014; Schütz et al., 2020; Unruh et al., 2019) were based on data collection on a 3T system, and six articles (Allen et al., 2004; Allen & Courchesne, 2003; Martineau et al., 2010; Müller et al., 2001; Müller et al., 2003; Müller et al., 2004) used a 1.5T system. Of the 16 records meeting the inclusion criteria, seven studies primarily investigated action execution (Allen et al., 2004; Allen & Courchesne, 2003; Lepping et al., 2022; Müller et al., 2001, 2003, 2004; Unruh et al., 2019) and five primarily investigated action observation (Cole et al., 2019; Grèzes et al., 2009; Marsh & Hamilton, 2011; Perkins et al., 2015; Schütz et al., 2020). Two studies focused on both action execution and observation (Dinstein et al., 2010; Martineau et al., 2010), and two studies focused on action imitation (Okamoto et al., 2014; Poulin‐Lord et al., 2014).

#### Number of citations

In total, the articles included in this review have been cited 1205 times (citations for each article are provided in Table 1). The majority of citations were generated from five studies (Allen et al., 2004; Allen & Courchesne, 2003; Müller et al., 2001, 2003, 2004), with 740 (61.4%) citations combined.

### Study participants

#### ASD participants

Across all studies, the total number of ASD participants was 233. Of those participants, 206 (88.4%) were male and 27 (11.6%) were female. Two studies by Allen and colleagues (Allen et al., 2004; Allen & Courchesne, 2003), and three by Müller and coworkers (Müller et al., 2001, 2003, 2004), respectively, appear to include the same participants. Correcting for assumed identical samples (Allen et al., 2004; Müller et al., 2003, 2004), gives 209 ASD participants with 183 males (87.6%) and 26 females (12.4%).

All ASD participants included in the selected articles had a clinical diagnosis from multidisciplinary assessment. All articles except Martineau et al. (2010) (93.8%) reported diagnosis based on DSM‐IV/V. Martineau et al. (2010) reported use of clinical assessment and journal reports prior to enrollment in the study. Across all articles, 11 (69%) reported use of autism diagnostic interview‐revised (ADI‐R), autism diagnostic observation schedule (ADOS), autism spectrum quotient (AQ), or childhood autism rating scale (CARS) as a complementary method for diagnosis (see Table 1).

Concerning IQ, all ASD participants had an average full scale IQ > 70. In total, 12 studies (75%) reported that IQ was assessed using Wechsler Adult Intelligence Scale (WAIS‐R, WAIS II‐III), Wechsler Intelligence Scale for Children (WISC II‐III, WISC‐R), or WASI‐II (see Table 1). Note that the method used to evaluate IQ was not reported in four studies (Cole et al., 2019; Dinstein et al., 2010; Perkins et al., 2015; Schütz et al., 2020).

#### NT participants

The total number of NT participants across all studies was 238, of which 196 (82.4%) were male, and 42 (17.6%) were female. With assumed identical samples removed (Allen et al., 2004; Müller et al., 2003, 2004), the total number of NT participants was 214 of which 173 were male (80.8%) and 41 were female (19.2%).

All NT participants had a full scale IQ > 70 (see Table 1). IQ evaluation method was reported in 12 studies (75%), and IQ was assessed using WAIS‐R, WAIS II‐III, WISC II‐III, WISC‐R, or WASI‐II. Four studies did not report evaluation method for IQ (Cole et al., 2019; Dinstein et al., 2010; Perkins et al., 2015; Schütz et al., 2020).

#### Sample sizes

As can be noted in Table 1, several of the included studies have small sample sizes (Allen et al., 2004; Allen & Courchesne, 2003; Dinstein et al., 2010; Grèzes et al., 2009; Martineau et al., 2010; Müller et al., 2001, 2003, 2004; Perkins et al., 2015). Despite the methodological issues associated with this, we have included all reported between‐group analyses to provide a complete overview of the current knowledge within the present, so far, limited field.

#### Age across studies

The majority (15 studies) reported mean ages spanning between 18 and 30 years old for both ASD and NT. One study (Marsh & Hamilton, 2011) reported a respective group mean age above 30 (no age ranges stated). Of the studies reporting age ranges (10 studies), seven studies included both children (<18 years) and adults. Note that five of these studies are the ones assumed to involve identical samples (Allen et al., 2004; Allen & Courchesne, 2003; Müller et al., 2001, 2003, 2004). Two of these studies involved one child per group (Allen et al., 2004; Allen & Courchesne, 2003), and three included one child in the ASD but not NT group (Müller et al., 2001, 2003, 2004). Since the number of children included in this review is low, and the mean age in all studies is >18 years, all studies have been included to evaluate effects associated with an adult population.

#### Handedness across studies

Three studies (Grèzes et al., 2009; Marsh & Hamilton, 2011; Perkins et al., 2015) did not report handedness of the participants, one (Martineau et al., 2010) employed exclusively right‐handed participants, and the remaining twelve studies involved both right‐, left‐ and/or mixed‐handed participants (although with the majority of participants preferring the right hand). Two studies (Poulin‐Lord et al., 2014; Unruh et al., 2019) reported laterality indices showing significantly stronger right‐hand preference in the NT compared with the ASD group.

### Studies focusing on action execution

#### Paradigms and procedures

Seven studies (Allen et al., 2004; Allen & Courchesne, 2003; Lepping et al., 2022; Müller et al., 2001, 2003, 2004; Unruh et al., 2019) focused exclusively on execution and two (Dinstein et al., 2010; Martineau et al., 2010) on execution and observation. The latter two studies reported separate results associated with either execution or observation, however, no results associated with a potential overlap between execution and observation were presented. Thus, the respective execution condition of these two studies is included in this section, and the observation condition is included under “Studies focusing on action observation” below. In addition, two studies investigated imitation where participants viewed images of hand gestures and then replicated these gestures (Okamoto et al., 2014; Poulin‐Lord et al., 2014). Findings related to this imitative hand execution are included in this section.

Out of these eleven studies involving execution, four different overarching task procedures, all involving hand actions, were used: Five studies (Allen et al., 2004; Allen & Courchesne, 2003; Müller et al., 2001, 2003, 2004) used button‐press tasks, two (Lepping et al., 2022; Unruh et al., 2019) utilized force adjustment (transducer press) compared with passive viewing, two studies administered a task involving hand postures (Dinstein et al., 2010; Martineau et al., 2010), and two (Okamoto et al., 2014; Poulin‐Lord et al., 2014) used hand/finger gestures according to instructions.

#### Behavior

Three studies (Dinstein et al., 2010; Martineau et al., 2010; Poulin‐Lord et al., 2014) did not report behavioral performance. Reports of behavioral performance focused on task execution. No study used a standardized motor assessment to measure general motor functioning during fMRI (see Table 2). Six studies (Allen et al., 2004; Allen & Courchesne, 2003; Lepping et al., 2022; Müller et al., 2001; Okamoto et al., 2014; Unruh et al., 2019) found no significant group differences in behavioral measures between the ASD and NT groups. Two studies (Müller et al., 2003, 2004) correspondingly reported no significant group differences, although both observed a significantly higher error rate in ASD relative to NT participants. Three studies (Lepping et al., 2022; Müller et al., 2001, 2003) found significantly increased variability in behavioral measures during fMRI in ASD compared with NT, and one (Unruh et al., 2019) noted greater variability in the ASD group, although failing to reach significance. Of those studies, Lepping et al. (2022) and Unruh et al. (2019) further reported no significant difference of an additional measurement of mean maximal voluntary contraction force between ASD and NT performed before the fMRI data collection.

#### Brain activity

**FIGURE 3:**
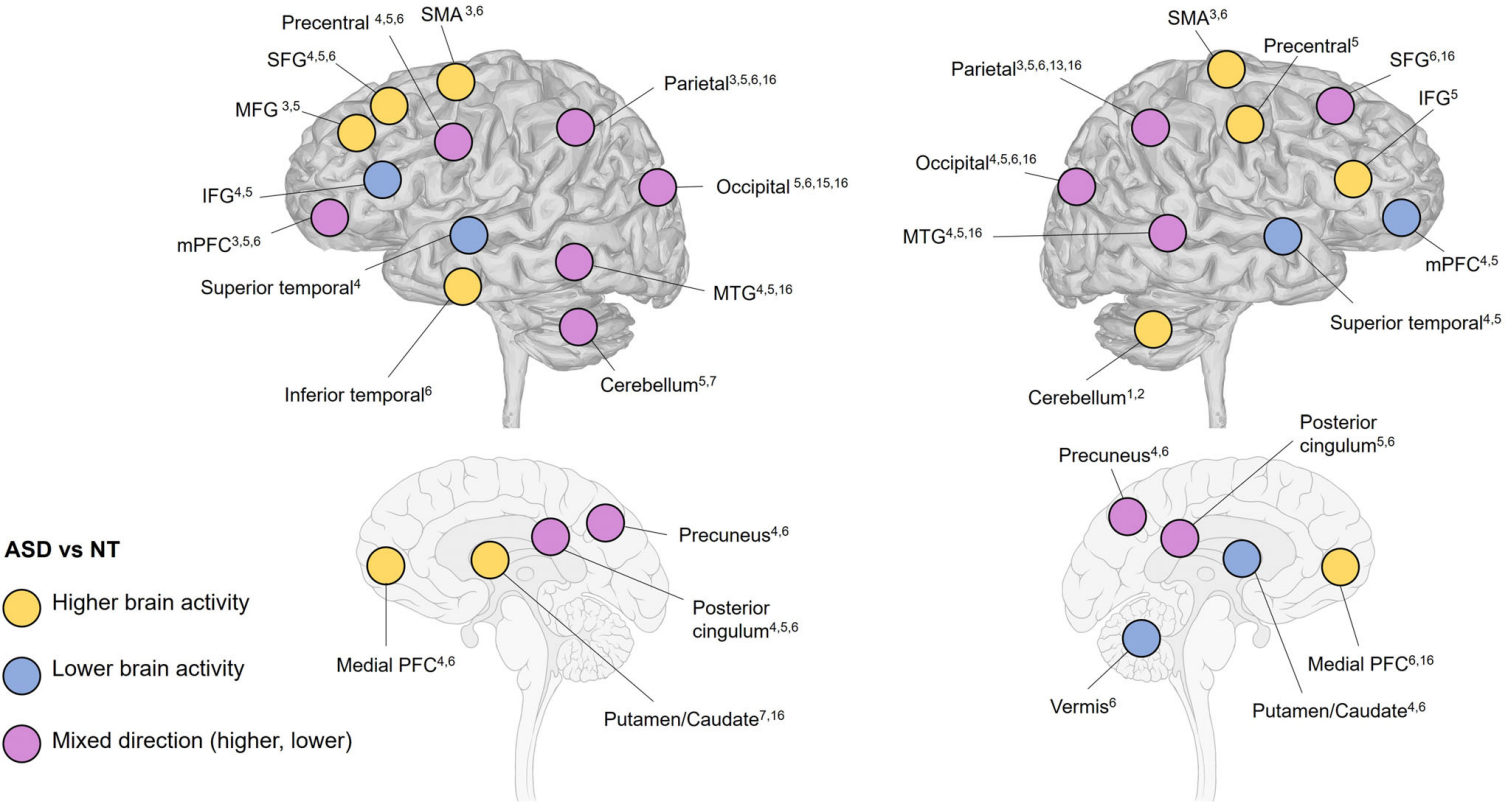
Synthesis of brain activation differences between the autism spectrum disorder (ASD) population and neurotypical (NT) population for the studies focusing on action execution. The numbers in superscript denote the specific publication reporting the result: Allen and Courchesne (2003)^1^, Allen et al. (2004)^2^, Lepping et al. (2022)^3^, Müller et al. (2001)^4^, Müller et al. (2003)^5^, Müller et al. (2004)^6^, Unruh et al. (2019)^7^, Cole et al. (2019)^8^, Grèzes et al. (2009)^9^, Marsh and Hamilton (2011)^10^, Perkins et al. (2015)^11^, Schütz et al. (2020)^12^, Dinstein et al. (2010)^13^, Martineau et al. (2010)^14^, Okamoto et al. (2014)^15^, and Poulin‐Lord et al. (2014)^16^. IFG, inferior frontal gyrus; MFG, middle frontal gyrus; MTG, middle temporal gyrus; mPFC, middle frontal cortex; SFG, superior frontal gyrus; SMA, supplementary motor area.

The fMRI results associated with activation differences for the ASD participants, as compared to the NT group, have been summarized separately, for each lobe, across both hemispheres. For a visualization of the results, see Figure 3 and Table S1.

Prefrontal activation differences were reported in several studies. Lower brain activity in the left IFG was reported in Müller et al. (2001) and Müller et al. (2003). Additionally, Müller et al. (2003) reported higher brain activity in the right IFG for ASD participants compared to NT participants. Müller et al. (2001, 2003) demonstrated higher brain activity in the left superior frontal gyrus (SFG) when comparing brain activity associated with execution of a simple motor task (pressing a button with index finger) for ASD compared with NT. Müller et al. (2004) further reported mixed activity in the SFG, with higher activity in the left hemisphere but lower in the right in ASD. Poulin‐Lord et al. (2014), however, reported higher activity in the right SFG in ASD in their visuo‐motor imitation task. Additionally, three studies observed higher activity in the left middle PFC (Lepping et al., 2022; Müller et al., 2003, 2004), while two studies (Müller et al., 2001, 2003) reported lower activity in the right middle PFC in ASD compared with NT participants. Moreover, one study showed higher activity bilaterally in the medial PFC in ASD participants (Müller et al., 2004). One additional study identified more engagement of the left medial PFC (Müller et al., 2001), and one study found higher activity in the right medial PFC (Poulin‐Lord et al., 2014) for ASD participants relative NT participants.

Related to engagement of premotor and motor areas, the bilateral supplementary motor area was reported to be more engaged in the ASD population in Lepping et al. (2022) and Müller et al. (2004). Moreover, a left‐sided activation of the precentral cortex in mixed directions in ASD was reported in three studies (Müller et al., 2001, 2003, 2004) with Müller et al. (2003) reporting mixed activation also in the right precentral cortex.

Parietal activity differences between ASD and NT were identified in both the right and left hemisphere. Two studies reported higher activity bilaterally in the superior parietal cortex in ASD (Lepping et al., 2022; Poulin‐Lord et al., 2014), one study reported higher engagement in the left superior parietal cortex (Müller et al., 2001). In contrast, Müller et al. (2003) observed lower activity bilaterally in the superior parietal cortex in ASD. Additionally, Müller et al. (2003, 2004) reported mixed activation bilaterally in the IPL. In the same region, Dinstein et al. (2010) noted lower right‐sided activity in ASD compared with NT participants. Moreover, two studies (Müller et al., 2001; Poulin‐Lord et al., 2014) observed higher activity bilaterally in the precuneus, while Müller et al. (2003, 2004) reported mixed activity.

Two studies reported higher right‐sided activity in the superior occipital cortex in ASD compared with NT participants (Müller et al., 2001, 2003). Two studies showed lower left‐sided activity in the middle occipital gyrus in ASD (Müller et al., 2004; Okamoto et al., 2014), one of them (Müller et al., 2004) also reporting lower right‐sided activity in this region. In addition, one study observed mixed activity in the middle occipital gyrus and lower activity in the inferior occipital gyrus in ASD (Poulin‐Lord et al., 2014). Moreover, four studies reported atypical activity (diverse directions) in the cuneus and lingual regions (Müller et al., 2001, 2003, 2004; Poulin‐Lord et al., 2014).

Müller et al. (2001, 2003) observed lower activity in ASD compared with NT participants in the right superior temporal lobe. In addition, Müller et al. (2001) found lower activity in the left anterior/superior temporal lobe. Several studies also reported effects in the middle temporal lobe, extending into the extrastriate body area. One study reported lower activity in ASD in the right middle temporal lobe, although higher activity in the left (Müller et al., 2001). In contrast, Müller et al. (2003) observed lower activity in the right middle temporal lobe and higher activity in the left in the ASD group. Moreover, Poulin‐Lord et al. (2014) found bilateral lower activity in the middle temporal lobe, while Müller et al. (2004) identified higher activity in the left inferior temporal lobe for ASD versus NT during action execution.

Five studies reported evident effects in the cerebellum when comparing ASD versus NT in simple (Allen et al., 2004; Allen & Courchesne, 2003; Müller et al., 2003, 2004) and more complex (Unruh et al., 2019) motor execution tasks. Two studies (Allen et al., 2004; Allen & Courchesne, 2003) found higher activity in the right superior lobule VIIa, while Unruh et al. (2019) found higher activity in the left lobule VIIb. Further, Allen et al. (2004) noted higher activity in the right anterior hemisphere, while Allen and Courchesne (2003) reported lower activity in the left anterior hemisphere of their ASD participants. One study reported lower brain activity in ASD participants compared with NT participants in bilateral vermis (Müller et al., 2004).

Additionally, Unruh et al. (2019) reported higher activity in the left putamen during execution of a force gripping task when comparing ASD and NT. Müller et al. (2001, 2004), however, found lower activity in the right putamen. Two studies further found differential activity in the caudate, one reporting lower activity in the right caudate (Müller et al., 2004) and one reporting higher activity in the left caudate (Poulin‐Lord et al., 2014). Moreover, three studies reported differential activity in middle/posterior cingulum, showing higher left‐sided activity (Müller et al., 2001), higher bilateral activity (Müller et al., 2004), and lower bilateral activity (Müller et al., 2003) in ASD compared with NT participants.

### Studies focusing on action observation

#### Paradigms and procedures

Five studies solely focused on action observation (Cole et al., 2019; Grèzes et al., 2009; Marsh & Hamilton, 2011; Perkins et al., 2015; Schütz et al., 2020) and two focused on execution and observation (Dinstein et al., 2010; Martineau et al., 2010), presenting separate data for the observation condition. The two studies focusing on imitation (Okamoto et al., 2014; Poulin‐Lord et al., 2014) did not present data exclusive for the observational element of the task and are thus not included in this section. Of the seven studies in total presenting an observation condition, four displayed videos involving actions of the hand/fingers (Cole et al., 2019; Marsh & Hamilton, 2011; Martineau et al., 2010; Perkins et al., 2015), one (Schütz et al., 2020) displayed human‐object interactions, and one (Grèzes et al., 2009) door opening actions. Notably, the remaining study (Dinstein et al., 2010) used still images of hand postures. As the present review is confined to naturalistic motor action, results regarding the observation condition in Dinstein et al. (2010) are thus excluded. In keeping with the execution tasks, all of the included studies with an observation condition utilized video clips involving hand actions, with the exception of one using full body movement (Grèzes et al., 2009).

#### Behavior

Reports of behavioral performance focused on outcomes of the observation task performed during fMRI (see Table 2). Four studies (Grèzes et al., 2009; Martineau et al., 2010; Perkins et al., 2015; Poulin‐Lord et al., 2014) did not report behavioral findings. One study (Cole et al., 2019) found no significant group differences between ASD and NT in task performance (mentalizing and nonmentalizing), although it was reported that level of autistic traits in both groups predicted behavior in the mentalizing task. One study (Schütz et al., 2020) found significantly slower reaction times across all conditions in the ASD group, and one study (Marsh & Hamilton, 2011) reported significant group differences in behavioral tasks outside of the scanner (mentalizing ability task and action comprehension task), with better performance among NT participants.

#### Brain activity

**FIGURE 4:**
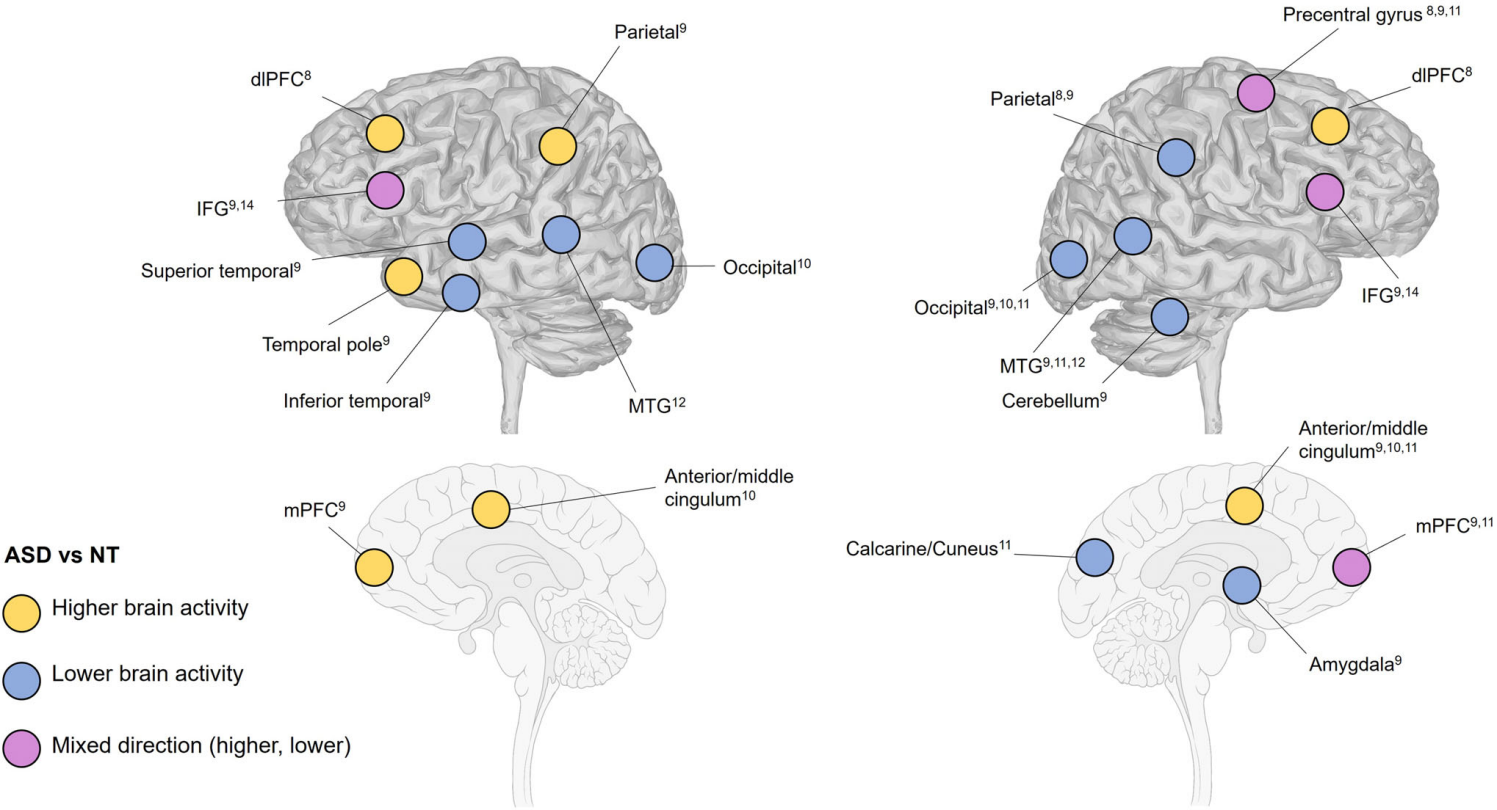
Brain activation differences between the ASD population and NT population in the studies focusing on action observation. The numbers in superscript denote the specific publication reporting the result: Allen and Courchesne (2003)^1^, Allen et al. (2004)^2^, Lepping et al. (2022)^3^, Müller et al. (2001)^4^, Müller et al. (2003)^5^, Müller et al. (2004)^6^, Unruh et al. (2019)^7^, Cole et al. (2019)^8^, Grèzes et al. (2009)^9^, Marsh and Hamilton (2011)^10^, Perkins et al. (2015)^11^, Schütz et al. (2020)^12^, Dinstein et al. (2010)^13^, Martineau et al. (2010)^14^, Okamoto et al. (2014)^15^, and Poulin‐Lord et al. (2014)^16^. IFG/dlPFC, inferior frontal gyrus/dorsolateral prefrontal cortex; mPFC, medial frontal cortex; MTG, middle temporal gyrus.

The fMRI results for reported activation differences for the ASD participants, relative to the NT participants, are summarized below. The reported results focus on each lobe separately, covering both hemispheres, for all studies. For a visualization of the results, see Figure 4 and Table S2.

One study reported lower activity bilaterally in the IFG during observation for ASD participants compared with NT participants (Grèzes et al., 2009) and one study reported higher bilateral activity in ASD in pars opercularis (Martineau et al., 2010). One study showed lower engagement (Grèzes et al., 2009) and one higher engagement (Perkins et al., 2015) of the right medial PFC. Grèzes et al. (2009) further found higher activity in the left medial anterior SFG in ASD, and one study reported higher activation in the dorsolateral PFC (Cole et al., 2019). In addition, one study (Grèzes et al., 2009) observed lower activity in the right middle frontal gyrus in ASD compared to NT participants.

Two studies showed lower activation in the right premotor/precentral cortex during action observation (Cole et al., 2019; Grèzes et al., 2009), while one showed higher activation in the same region (Perkins et al., 2015). One additional study reported lower activity in the left subcentral gyrus (Marsh & Hamilton, 2011).

Cole et al. (2019) focused on task‐related connectivity and reported, as opposed to the NT group, no significant connectivity between a mentalizing and nonmentalizing task in the bilateral IPL (as part of the MNS) in the ASD group. In addition, Grèzes et al. (2009) showed lower activity in ASD in a right superior inferior parietal region. Notably, Perkins et al. (2015) also reported a difference (of unclear direction) in parietal cortex bilaterally in ASD, although failing to reach significant voxels compared with NT.

One study reported bilateral effects related to lower activity in the middle temporal gyrus/extrastriate body area (MTG/EBA) (Schütz et al., 2020). Two additional studies reported lower activation in this brain region in the right hemisphere (Grèzes et al., 2009; Perkins et al., 2015). Grèzes et al. (2009) further showed bilateral lower activity in the inferior temporal gyrus and differential activity in the temporal parietal junction, with lower activity in the right and higher in the left hemisphere. Grèzes et al. (2009) also observed lower activity in the right superior temporal sulcus, higher activity in the right temporal gyrus and higher activity in the left temporal pole/insula in ASD compared to NT participants. Finally, Grèzes et al. (2009) also found lower activity in the right fusiform gyrus (extending into the cerebellum).

One study identified lower brain activity in the ASD group, relative the NT group, in a left parieto‐occipital region (Marsh & Hamilton, 2011). Similarly, one study reported lower activity in the right calcarine gyrus and the right cuneus for ASD participants during observation of hand‐based gestures (Perkins et al., 2015). Grèzes et al. (2009) also showed lower activity in the right lingual gyrus in ASD.

Regarding the cingulum, three studies focusing on observation noted group differences in the middle/anterior cingulum (as opposed to the middle/posterior cingulum in studies focusing on execution). Grèzes et al. (2009) reported right‐sided lower activity in the cingulum, whereas Perkins et al. (2015) found higher activity in ASD compared with NT. One study (Marsh & Hamilton, 2011) observed bilateral lower activity in the cingulum extending into the motor area. Finally, one study (Grèzes et al., 2009) reported lower activity in the right amygdala related to perception of fearful versus neutral actions.

### Studies focusing on action imitation

As described in the sections above, two studies explored action imitation in terms of observing and replicating hand gestures (Okamoto et al., 2014; Poulin‐Lord et al., 2014). Neither of these studies reported fMRI results related to the observational element of the task part. They did however report the executional elements of the imitation activity. Thus, henceforth the two imitation studies are sorted under action execution in the summary below.

### Overlap between studies focusing on action execution and observation

**FIGURE 5:**
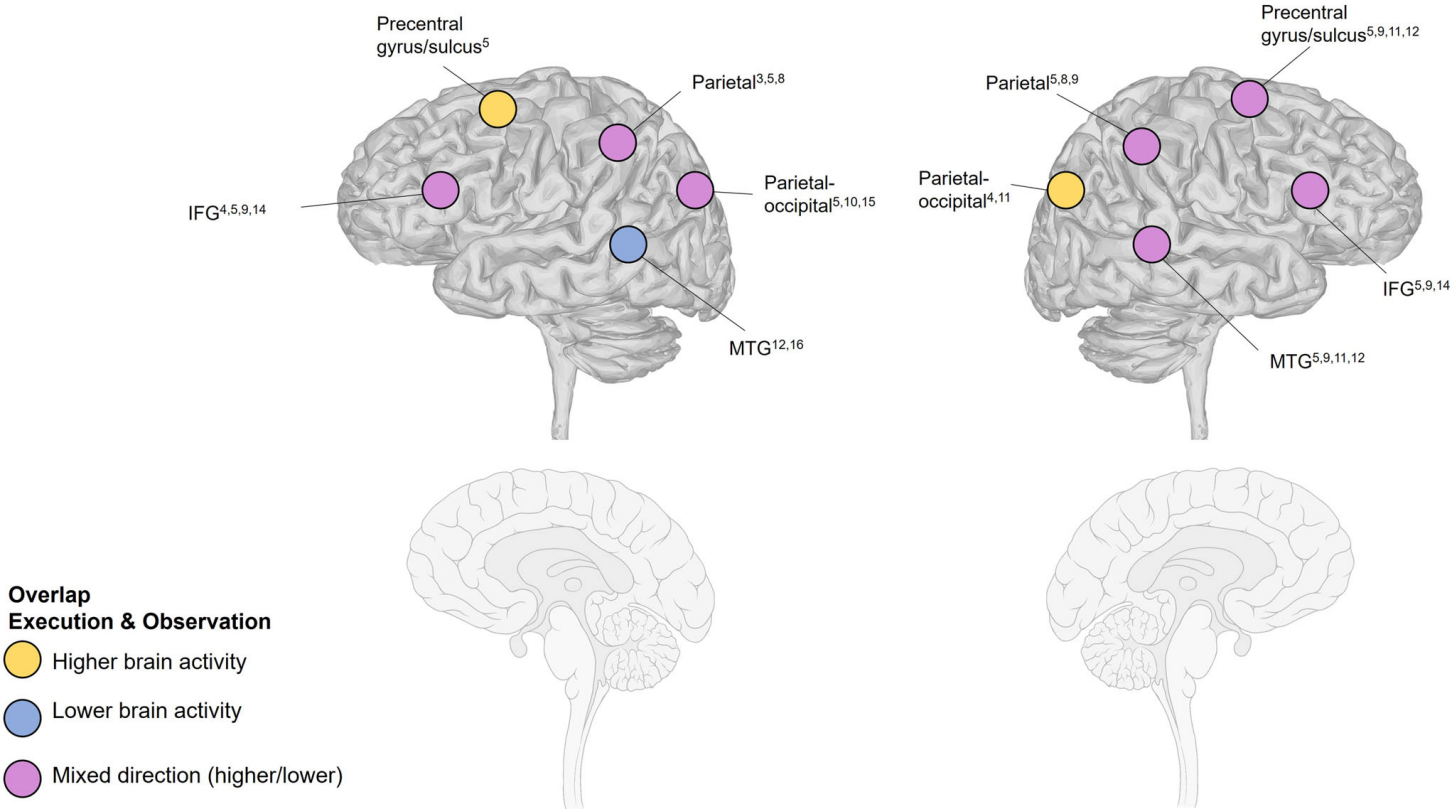
The overlap in brain activations in studies focusing on action execution and/or action observation. The numbers in superscript denote the specific publication reporting the result: Allen and Courchesne (2003)^1^, Allen et al. (2004)^2^, Lepping et al. (2022)^3^, Müller et al. (2001)^4^, Müller et al. (2003)^5^, Müller et al. (2004)^6^, Unruh et al. (2019)^7^, Cole et al. (2019)^8^, Grèzes et al. (2009)^9^, Marsh and Hamilton (2011)^10^, Perkins et al. (2015)^11^, Schütz et al. (2020)^12^, Dinstein et al. (2010)^13^, Martineau et al. (2010)^14^, Okamoto et al. (2014)^15^, and Poulin‐Lord et al. (2014)^16^. IFG, inferior frontal gyrus; MTG, middle temporal gyrus.

One main purpose of the synthesis of results was to provide a description of brain areas with overlapping activity for different kinds of motor action (execution, observation, and imitation). Note that the overlapping effects have been drawn from proximity of reported brain areas and are not based on a metanalytic procedure (see Table S3 for specific coordinates). Ten brain regions located in the left and right hemisphere were found to overlap between the execution (including imitation) and observation conditions (see Figure 5). Three studies reported bilateral activation of the IFG (Grèzes et al., 2009; Martineau et al., 2010; Müller et al., 2003), but the direction of effects varied. Three studies showed lower activation in the left IFG for ASD compared with NT (Grèzes et al., 2009; Müller et al., 2001, 2003), and one higher activation (Martineau et al., 2010). In the right IFG, two studies (Martineau et al., 2010; Müller et al., 2003) found higher activity, and one study (Grèzes et al., 2009) found lower activity in ASD compared with NT participants.

Four studies demonstrated differential activation of the precentral gyrus/SMA, one with higher brain activity in the right precentral gyrus in the ASD group (Perkins et al., 2015), one with higher activity bilaterally (Müller et al., 2003), and two with lower right‐sided activity in the ASD group compared with the NT group (Grèzes et al., 2009; Schütz et al., 2020).

Four studies (Cole et al., 2019; Grèzes et al., 2009; Lepping et al., 2022; Müller et al., 2003) reported differential activation of parietal regions, all extending into and/or neighboring the IPL. Müller et al. (2003) showed higher bilateral parietal activation in the ASD group compared with the NT group, Lepping et al. (2022) reported lower left‐sided parietal activation in ASD, Cole et al. (2019) showed lower parietal activation bilaterally in ASD, and Grèzes et al. (2009) reported lower activation in the right superior IPL.

Engagement of the MTG was reported in five studies covering both hemispheres (Grèzes et al., 2009; Müller et al., 2003; Perkins et al., 2015; Poulin‐Lord et al., 2014; Schütz et al., 2020). Two studies (Grèzes et al., 2009; Perkins et al., 2015) reported lower activation exclusively in the right MTG, and one (Poulin‐Lord et al., 2014) exclusively in the left MTG. One study demonstrated higher activity within the ASD group exclusively in the right MTG (Müller et al., 2003). One study reported lower activation in the bilateral MTG in ASD compared with NT (Schütz et al., 2020).

Five studies (Marsh & Hamilton, 2011; Müller et al., 2001, 2003; Okamoto et al., 2014; Perkins et al., 2015) reported atypical activation in the parietal‐occipital cortex. Two studies (Müller et al., 2001; Perkins et al., 2015) reported higher activity in the right hemisphere, two studies (Marsh & Hamilton, 2011; Okamoto et al., 2014) showed lower activity in the left hemisphere, whereas Müller et al. (2003) demonstrated higher activity in the left hemisphere.

## DISCUSSION

In this review, we aimed to summarize the collected fMRI findings over the last two decades related to regional brain engagement subserving naturalistic action execution, observation, and imitation in adults with ASD compared with NT adults. An additional aim was to describe the overlap between action execution and observation across regions where adults with ASD display atypical activity based on the included studies. As expected, with emphasis on manual actions, we found that adults with ASD mainly showed activations in similar locations as NT adults, such as frontoparietal and occipitotemporal cortical regions, the cerebellum and MNS/AON regions, although deviating in magnitude and/or direction. Moreover, the activity overlaps between action execution and observation in autistic adults are consistent with a suggested neural basis for challenges in motor‐based understanding of others' action and intentions.

Notably, however, the limited quantity of publications and countries/research institutions represented suggest that the research field is challenging and still emerging. As judged by the lack of an increasing trend line in annual publications (Figure 2), the field has not expanded as typically seen in other types of fMRI research across a similar time period (Stelzer et al., 2014). The earliest studies included in this review (Allen et al., 2004; Allen & Courchesne, 2003; Müller et al., 2001, 2003, 2004) have some methodological shortcomings compared with current standards for validity and generalizability in fMRI research. This may have exaggerated some of the overlaps reported in this review. Still, given that the collected findings of these pioneering articles harmonize with those from more recent studies, their methodological limitations do not undermine the general conclusions that can be drawn from this review.

There was a clear majority of male ASD participants with an average IQ across the studies included in this review. Thus, it is not obvious that the overview presented here can be readily translated to female presentations of ASD and/or individuals with concurrent intellectual disability. Moreover, even if a few child/adolescent participants are included in the reported study samples, it is not possible to disentangle effects associated with adults per se from those of children. Notably, however, one study involving both child and adult participants (Lepping et al., 2022) found increased connectivity with age in the cerebellum in the ASD compared with NT group. Thus, age may impact connectivity differences between larger brain networks in ASD versus NT (Cerliani et al., 2015).

Regarding IQ, cognitive control processes are functionally related to motor behavior and associated brain activity (Serrien et al., 2007), suggesting potentially important links between cognition and action. Unfortunately, none of the studies included in this review used IQ as a covariate or controlled for IQ (beyond including a comparison group) in their analyses of brain activations. In addition, none of the studies reported comorbidity within their ASD samples, which may be important to consider given the high prevalence of comorbidity in ASD populations. Thus, it is unclear whether the reported findings are specific for ASD or shared between various neuropsychiatric conditions. Indeed, specific patterns of connectivity related to social skills have been shown in youth with ASD (Jayashankar et al., 2023) pointing toward the importance of continued research on this topic.

The studies included a majority of right‐handed participants with ASD. Although the reported proportion of right‐handedness was similar in the ASD and NT groups in most included studies (possibly due to matching), the two studies reporting laterality indices for handedness (Poulin‐Lord et al., 2014; Unruh et al., 2019) observed that ASD participants were not as strongly right‐handed as NT participants. This is in keeping with findings of lower rates of right handedness in individuals with ASD (Escalante‐Mead et al., 2003).

Of the studies reporting behavioral findings, most did not observe significant group differences in task performance. This is possibly due to the mild to moderate form of autism characterizing the autistic participants. Although, where evident differences or trends in behavioral measures were noted, they consistently pointed toward autism being accompanied by less optimal performance. In line with findings of impaired fine motor skills (Sacrey et al., 2014), this was particularly apparent during action execution. Notably, the motor tasks employed in the studies were typically goal‐directed. The difficulties displayed may thus be related to impaired predictive motor behavior in ASD, including accentuated variability (Bäckström et al., 2021; Zheng et al., 2019).

Regarding action execution, some brain regions are frequently reported as atypically engaged in ASD individuals. One of these is the cerebellum, well‐known to vary in structure and function in ASD and related to deviations in motor behaviors (Butera et al., 2023; Thabault et al., 2022). Other repeatedly reported regions include parts of the fronto‐parietal, temporal and motor cortices, which are involved in several different action‐related activities, such as storage of motor representations (Grafton & Hamilton, 2007; Leisman et al., 2016) sensorimotor integration (Buneo & Andersen, 2006) and motor execution (Hardwick et al., 2018). Notably, these cerebellar and cortical deviations are consistent with recent studies of brain activations during action execution in ASD using other brain imaging techniques, such as combined functional near infrared spectroscopy (fNIRS) and electroencephalogram (EEG) (c.f., Su et al., 2023). Thus, this review corroborates that difficulties with motor representations, planning and performance commonly observed in ASD seem to be paralleled by atypical brain engagement in regions known to subserve such aspects of naturalistic actions. The types of goal‐directed actions described in the reviewed studies typically engage cortico‐striatal pathways, known to be aberrant in individuals with ASD (e.g., Balleine & O'Doherty, 2010). Here, this suggested atypicality was reflected by either higher or lower striatal engagement (putamen/caudate) in ASD across the hemispheres. Notably, the studies showing higher striatal activity (Poulin‐Lord et al., 2014; Unruh et al., 2019) used a more complex form of naturalistic action task. Thus, more complex motor activities may be required to produce and/or reveal atypically higher striatal engagement in ASD. A caveat to the differential activation between ASD versus NT during action execution is that it could be complicated by comorbidity with other neurodevelopmental disorders. For example, in the case of ASD and cooccurring developmental coordination disorder, atypical brain activity may not necessarily be ASD‐specific (Kilroy et al., 2021).

Regarding action observation, this review identified recurrent reports of deviating brain activity in brain regions ascribed to the MNS/AON (bilateral IFG, MTG, IPL, precentral gyrus/SMA, and parieto‐occipital cortex). The precentral gyrus is an important brain region for accessing stored motor representations as well as understanding actions of others (e.g., Ogawa & Inui, 2011). Speculatively, the deviating activation reported in the studies included in this review may indicate problems with accessing stored motor representations. The bilateral IFG is implicated in processing the actions of others (Kilner et al., 2009), whereas the posterior MTG has been linked to processing of biological motion (Caspers et al., 2010). It has also been suggested that the MTG is involved in the process of matching others actions with one's own stored motor representations, via visual input received from the occipito‐parietal cortex (Mahon & Almeida, 2024). Thus, the underactivations in MTG and occipital regions consistently reported in the ASD population in this review may indicate atypical engagement of brain regions that are critical for accessing and matching ones stored motor representations as well as regulating visual input during action observation. Notably, one study (Grèzes et al., 2009) used a door opening task (full body movement) as opposed to hand actions in the other observation studies. This study also reported more widespread engagement of brain regions than the other studies (see Table S3), in particular regarding temporal regions.

The action execution‐observation activity overlaps also suggest deviating activity in the MNS/AON (bilateral IFG, MTG, precentral, and parieto‐occipital brain areas) in ASD. It has been suggested that motor impairments in ASD arise from abnormal recruitment of brain networks involved in motor behavior, including fronto‐parietal and occipito‐temporal regions subserving motor planning and/or predictive control (Casartelli et al., 2018; Gallivan et al., 2016; Säfström & Domellöf, 2018). The activity overlaps presented here are in keeping with these suggestions. Moreover, the IPL is engaged in planning and control of movements as well as sensory‐motor integration (Buneo & Andersen, 2006). Combined, deviating activation during both execution and observation in the IFG and IPL could reflect difficulties accessing representations needed for both action execution and for matching the representation of an observed action with stored experience of own action. Although, another interpretation could be that both the IFG and premotor regions identified in the overlap comparison have dissociable roles for execution and observation of hand actions in NT individuals (e.g., Press et al., 2012).

Sensory‐motor performance variability in ASD has been linked to inefficient planning and feedforward control, possibly related to the structural and/or functional integrity of the cerebellum and basal ganglia (Foster et al., 2020). Some support for this notion is found in the atypical brain activations in ASD presented here. Reported brain activation variability in ASD, however, appears to be widely distributed throughout the brain. Thus, how individual variability in brain activity in relation to action in ASD may contribute to sensory‐motor difficulties warrants further study.

It should be stressed that the noticeable differences between studies in terms of employed tasks may also be a source of variability in brain activations reported here. As judged by this review, type of task appears to generate different brain activation patterns to a certain degree. For example, as discussed above, it may be that more complex action execution tasks (e.g., finger force adjustment) generate higher subcortical brain activity. In action observation tasks, the use of full body movement stimuli may produce more engagement of temporal regions than hand gestures. Still, reported effects sometimes differed between studies using similar tasks, suggesting that task‐activity relations are multifaceted and require specific investigation.

## CONCLUSIONS

While based on a, to date, modest amount of fMRI studies of naturalistic action in autistic individuals, this review corroborates that impairments in action execution, observation, and imitation are related to atypical activation patterns in underlying, partly overlapping, brain regions. Considering that motor problems have been suggested as a core symptom in ASD, it is important to further pursue this line of research to provide a reliable scientific basis for clinical recommendations and interventions. Also, given that motor impairments may be connected to the social manifestations in ASD, an increased understanding of the underlying mechanisms may also unveil important neural foundations for the broader ASD symptomatology. This review has focused on autistic adults compared with NT adults. Still, the effects reported here could look differently if children/youth were considered (e.g., Cerliani et al., 2015; Kilroy et al., 2021). Therefore, an overview on action execution and observation in children/youth with ASD alongside adult data is warranted.

## Supporting information


**Table S1.** Significant group differences between ASD and NT reported in studies focusing on action.

**Table S2.** Significant group differences between ASD and NT reported in studies focusing on action observation.

**Table S3.** Overlapping activation areas between action execution and action observation.

## Data Availability

Data sharing not applicable to this article as no datasets were generated or analysed during the current study.
